# Evaluating the Effects of a Mobile Obstetric Emergency System on Healthcare Providers’ Communication and Relationships in Bong County, Liberia

**DOI:** 10.3390/healthcare14121738

**Published:** 2026-06-16

**Authors:** Tiffany Lin, HaEun Lee, Karina Paredes, Alisha Keshwani, Joseph Sieka, Jody R. Lori

**Affiliations:** 1 School of Nursing, University of Michigan, Ann Arbor, MI 48109, USA; haeunlee@umich.edu (H.L.); karinaa@umich.edu (K.P.); akesh@umich.edu (A.K.); jrlori@umich.edu (J.R.L.); 2 College of Health Sciences, University of Liberia, Capitol Hill P.O. Box 10-9020, Monrovia 1000, Liberia; jmatusieka@gmail.com

**Keywords:** maternal health, healthcare system innovations, obstetric referral systems, digital health, mobile health (mHealth), LMIC

## Abstract

**Background/Objectives**: Maternal mortality remains disproportionately high in low- and middle-income countries, where ineffective referral systems and a lack of infrastructure contribute to delays in emergency obstetric care. In sub-Saharan Africa, referrals are largely conducted via paper, often resulting in lost documents and limited follow-up. Mobile health (mHealth) offers a promising solution by enabling real-time, bidirectional communication. This study aimed to examine how the Mobile Obstetric Referral Emergency System (MORES), a WhatsApp-based referral platform piloted in 20 rural health facilities and two district hospitals in Bong County, Libera, influences healthcare providers’ communication, collaboration, and relationships. **Methods**: A mixed-methods design was used. Ninety one (N = 91) providers completed demographic and Trust and Teamwork surveys. Of the 91 providers, 35 providers from rural health facilities and 56 providers from district hospitals participated in a 10-question survey and individual interviews. **Results**: Survey results indicated high levels of mutual respect, confidence, and teamwork perceived by both the rural health facility and district hospital providers. Qualitative data further expanded on the quantitative results showing the MORES intervention enhanced the timeliness and accuracy of referrals, supported problem-solving between facilities, and fostered shared goals, mutual respect, and knowledge exchange. **Conclusions**: Providers perceived the MORES to be associated with increased collaboration and continuity of care, as well as a feasible, low-cost, and sustainable intervention to improve obstetric referral systems in low-resource settings.

## 1. Introduction

Sub-Saharan Africa is disproportionately burdened by maternal morbidity, with over 70% (182,000) of the world’s maternal deaths in 2023 occurring in this region alone [1]. A significant contributor to the high maternal mortality rate is the frequent delays during emergency obstetric referral from the rural health facility to a district hospital. Rural health facilities (RHFs) in Africa are often equipped to perform basic emergency obstetric and newborn care (BEmONC), including administration of parenteral antibiotics, uterotonics, and anticonvulsants; removal of retained placenta; assisted vaginal delivery; and basic newborn resuscitation [2]. District hospitals are equipped to provide comprehensive emergency obstetric and newborn care (CEmONC), including blood transfusions and cesarean sections as well as the seven BEmONC functions [2]. As such, RHFs are meant to refer critical obstetric patients that require CEmONC services to the district hospital for additional care. In the process of referral, delays can occur that contribute to adverse maternal and newborn outcomes. For example, a study of 712 maternal deaths across 427 healthcare facilities in Mozambique found delays in the referral process contributed to 40.4% of the maternal mortalities [3].

Liberia is significantly affected by delays in the obstetric referral system, which can be compounded by ineffective communication between RHFs and hospitals [4]. Many district hospitals in Liberia report delayed or missed referrals from RHFs [5,6]. A study that examined 62 Liberian healthcare facilities and their referral system found only 19% of the referring facilities contacted the referral hospital prior to the referral [7]. Although 82% of facilities reported giving written referral forms to their patients to bring to the hospital, only 40% of the hospitals reported receiving referral forms [7]. A study of 458 Liberian healthcare centers found that only 40% were ready to make an emergency referral at any given moment, highlighting the lack of emergency preparedness and limited capabilities to refer patients [8]. Meanwhile, RHFs report a lack of feedback from district hospitals, which creates a barrier to follow-up care [5,6]. Ultimately, the current referral system makes continuity of care difficult to achieve due to interruptions in communication between healthcare facilities.

Mobile health (mHealth) shows great promise in promoting effective referral communication as it allows for quick bidirectional communication through mobile devices, eliminating the need for paper referrals and addressing the limited one-way communication [9,10]. WhatsApp is a digital messaging platform that has been piloted in studies as a health education and compliance tracking tool in LMICs [11,12,13]. WhatsApp provides a low-cost, fast, and secure technology with end-to-end encryption to facilitate clinical communication, enhance learning at the community level, and improve maternal and newborn outcomes [14]. There is a lack of research regarding the influence that mHealth intervention has on collaboration and communication between providers in LMICs.

Our parent study established an obstetric referral system using the WhatsApp messaging platform in rural Bong County, Liberia, to facilitate digital referrals between the district hospitals and the RHFs [15]. From March 2022 to March 2023, the MORES intervention was piloted across 20 RHFs and two district hospitals with 102 healthcare providers [15]. The MORES intervention provided RHF and hospital providers with messaging templates and brief training regarding the use of the MORES. The training aimed to strengthen inter-facility referrals through RHF follow-ups with hospital providers, allowing for bidirectional, closed-loop communications [2,15]. For the study, reporting templates were designed to ensure consistent, bidirectional communication. No patient names were used, and providers were assigned ID numbers. Data were shared only within the individual WhatsApp group between the RHF and district hospital staff participating in the research [2]. Our earlier studies established the feasibility and acceptability of WhatsApp for obstetric provider communication in rural Liberia [16,17]. Widespread existing use among providers minimized training needs, and low-bandwidth requirements suited rural connectivity limitations. Privacy and data governance were addressed through staff training, clear protocols, and anonymous data exchange, with full clinical data transferred via hand-carried paper records accompanying the patient. This separation of rapid communication from sensitive data transmission supports health worker acceptability and policy uptake by aligning with data protection principles without requiring new infrastructure. WhatsApp increases connectedness for an isolated workforce and reduces delays to skilled obstetric care in resource-constrained settings [16,17]. The details regarding the MORES implementation are provided elsewhere [2,16].

The main findings from the pilot study include that the MORES was effective in reducing the decision-to-delivery interval in obstetric emergencies [18]; women had higher odds of undergoing cesarean sections and newborns had lower odds of being non-vigorous at birth compared to before MORES implementation [2]. Healthcare providers perceived the MORES to have increased their attentiveness towards patients and sense of responsibility towards their care, making them more comfortable with acknowledging their limitations and referring if deemed necessary [15]. However, there is a lack of research on the influence of the MORES on providers’ perceived trust and collaboration across facilities.

### Theoretical Framework

This paper is a follow-up study that utilizes relational coordination theory to explore the types of communication and collaboration fostered by the MORES [3]. Relational coordination theory explores how communication patterns and relationship qualities intersect to influence the fulfillment of tasks amongst a team in a complex, interdependent setting, such as a network of healthcare centers [19]. Effective collaboration and coordination of work amongst co-workers is based on a mutual sense of respect, trust and shared goals that serve as motivation to facilitate timely, accurate and problem-solving communication [19]. Likewise, communication patterns that emphasize promptness and precision foster a deeper sense of respect and trust between workers, enabling them to complete their assigned tasks more efficiently [19].

## 2. Materials and Methods

A convergent mixed-methods design was used in this study. We followed up one year after implementation of the MORES intervention to conduct surveys and interviews among 91 providers across 20 RHFs and two district hospitals that participated in the original MORES study. Participants were recruited through flyers posted at the RHFs and district hospitals. Eligible participants were 18 years or older and currently employed as a nurse, midwife, and/or physician assistant at the selected facilities. A demographic survey, as well as a Trust and Teamwork Survey consisting of 10 questions, was collected from all participants. Of those who completed the survey, individual interviews (IIs) were conducted with 35 RHF providers and 56 hospital providers.

### 2.1. Setting

This study took place in Bong County, Liberia. Liberia has the sixth highest maternal mortality ratio in the world, with over 652 women dying per 100,000 live births due to pregnancy-related causes [20]. Bong County is the third most populous county in Liberia, with a population of around 329,000 people [21]. In 2019, there were 15,231 facility births attended by skilled healthcare providers reported in the county [2]. Most of these births (81.16%) occurred at a rural clinic with a midwife or nurse. If complications occur, needing an emergency cesarean section, RHFs will refer the patient to a district hospital [7]. Prior to MORES, most facilities conducted paper referrals that would often get lost in transit [15]. There is a prominent lack of ambulances, with only two ambulances for the entire county [15]. Bong County was selected as the study site due to it being the site of the pilot intervention.

### 2.2. Data Collection Tools

The survey included demographic parameters such as participants’ age, occupation, years in their profession, years spent at their current location, and whether they were part of the team when MORES was first implemented or not. A Trust and Teamwork Scale was also included, asking the participants to rate their agreement with various statements on a Likert scale of 1 to 5, with 1 indicating not confident/strongly disagree and 5 indicating very confident/strongly agree. The Trust and Teamwork Scale was first developed and used by members of our team and tested for validity to assess trust and teamwork amongst traditional birth attendants and midwives in Liberia [22]. Our research team further modified it to reflect the needs of this study and to examine trust and teamwork between licensed providers. Cronbach’s alpha for the revised tool was 0.65, with the lowest correlation for the last two variables related to the MORES intervention (“I feel like the use of WhatsApp improved referral communication” and “Receiving patient feedback improves my clinical skills/Receiving advanced knowledge of a referral helps me prepare for the patient’s arrival”), which is expected. While falling slightly below 0.70, it is within an acceptable range for exploratory research, and the scale is interpreted with appropriate caution. Questions were split into two sections that assessed trust and teamwork. Questions regarding trust included “I respect the providers from the [RHF/hospital]?” and “I trust the clinical judgment of the providers in the [RHF/hospital]?”. Questions regarding teamwork included “I feel like part of a healthcare team.” and “I feel like the use of WhatsApp improves referral communication.” Certain questions had two different versions based on the facility the participant works at. For example, RHF providers were asked to rate “Receiving patient feedback improv[ed] [my] clinical skills?” and hospital providers were asked to rate “Receiving advanced knowledge of a referral help[ed] [me] prepare for the patient’s arrival?”.

The IIs were conducted using a semi-structured interview guide of 24 open-ended questions. Probing questions asked about reasons for continued or discontinued use of MORES, benefits and drawbacks of MORES, and effects on collaboration and trust between healthcare workers. These guides were also used in the MORES pilot study and were developed in an iterative process by obstetric experts in our team representing both the University of Michigan and University of Liberia.

### 2.3. Data Collection

Three research assistants (RAs) from the University of Liberia completed pre-fieldwork training to provide an overview of the study and rigorous training on interview techniques, ensuring the reliability and validity of the data, data recording, and ethical considerations. A non-judgmental environment and indirect questioning techniques were used to decrease respondent bias [18]. Healthcare workers from healthcare facilities that participated in the pilot intervention were invited to participate in the study via flyers and word-of-mouth. Interested individuals reached out to the RAs to arrange meeting times regarding their participation in the study. The consent form explained all data would be de-identified and would not affect their employment status, and any questions could be skipped if they wished. The IIs occurred over a span of 45 to 60 min. Audio from the IIs was recorded via a tape recorder, and notes were taken by RAs. All interviews were conducted in English, as English is Liberia’s official language and providers were fluent [20].

### 2.4. Data Analysis

The audio recordings were transcribed verbatim by paid transcriptionists and stored in a secure Dropbox folder accessible only to researchers. All identifying information was removed from the transcripts. All quantitative data were analyzed using descriptive statistics, chi-square tests for categorical variables and independent *t*-tests for continuous variables using Stata (18.0). For the Trust and Teamwork Survey responses, we captured the mean (SD), median (IQR), and ran exploratory inferential tests, Mann–Whitney U test, given the nature of Likert-scale data and the skewed response distribution observed.

All qualitative data were imported into Dedoose 9.0.107 and analyzed through inductive coding and constant comparative methods [23]. Inductive coding involves sorting data into certain codes that fall under overarching themes pertaining to the relational communication framework, and analyzing the patterns and connections formed by the codes to generate a more nuanced understanding of the participant’s viewpoints [24]. Constant comparative method and pattern coding was used to identify common themes and modify instruments and procedures [25]. Three researchers (TL, HL, and KP) independently read through a batch of 10 of the same transcripts each to develop the codebook. During weekly team meetings, any disagreements in coding were discussed until consensus was met among all researchers. Once the codebook was finalized, each researcher coded through another batch of 10 interviews and continued to meet weekly to discuss the coded segments. All researchers had to agree on the validity of the coded segment, and discussions were held if inconsistencies arose. Additional codes were developed and eliminated in an iterative process. Upon completing the coding for all transcripts, two researchers went over the earlier process, and earlier coded interviews were re-coded at the end to ensure all codes were accurately applied. Interviews continued until thematic saturation was achieved. Saturation was determined by the researchers in the field, who conferred and agreed that no new emerging patterns or themes were emerging from the interviews.

## 3. Results

### 3.1. Demographics

This study included 91 healthcare providers, 35 (38.45%) from 20 rural health facilities and 56 (61.53%) from two district hospitals (CB Dunbar and Phebe) (Table 1). The sample was predominantly nurses and midwives and a few physician assistants. Providers at district hospitals generally had more work experience in their fields, 10.4 years compared to 8.3 years for RHF providers. Most participants had their own personal smartphones (96.7%) and used the WhatsApp messenger application outside work (86.8%). Additionally, most participants who were part of the pilot MORES intervention (92.3%) still worked at the same facility (86.8%) and reported continued use of the MORES intervention (82.4%). “Prefer not to answer” responses were treated as missing data and excluded from the denominator when calculating percentages for each variable (i.e., pairwise deletion was used).

### 3.2. Trust and Teamwork Survey

The healthcare providers’ overall perceptions of trust and teamwork between two types of healthcare facilities are presented in Table 2. All ratings of the questions averaged higher than four on the Likert scale, indicating that providers strongly agreed with the positive statements about trust and teamwork. The only question that was rated low was the inversely worded question: “I feel like providers at the hospital/rural health facility are an obstacle to my work.” By rating the question very low (1.8 ± 0.4), providers strongly disagreed with the statement and viewed the other members of the healthcare team to be assets to their clinical work.

Both RHF and hospital providers rated their respect for providers at other facilities high at 5.0 (IQR = 4.0–5.0), but providers from the hospital reported respecting their counterparts more at 5.0 (IQR = 4.0–5.0) compared to RHF providers, 4.0 (IQR = 4.0–5.0). Hospital providers also felt they received more respect from the RHF providers (5.0; IQR = 4.0–5.0) and RHF providers had significantly higher confidence in the hospital providers’ skills (4.0; IQR = 4.0–5.0). Overall, providers rated their trust in each other’s clinical judgment high at 4.0 (IQR = 4.0–5.0). Both groups felt that they were valued as a member of the healthcare team (5.0; IQR = 4.0–5.0). Most RHF providers perceived that receiving feedback improved their clinical skills (5.0; IQR = 4.0–5.0), and all hospital providers perceived that receiving knowledge of the referral ahead of time helped them prepare for the patient (5.0; IQR = 4.0–5.0).

### 3.3. Themes from Participant Interviews

**Figure 1:**
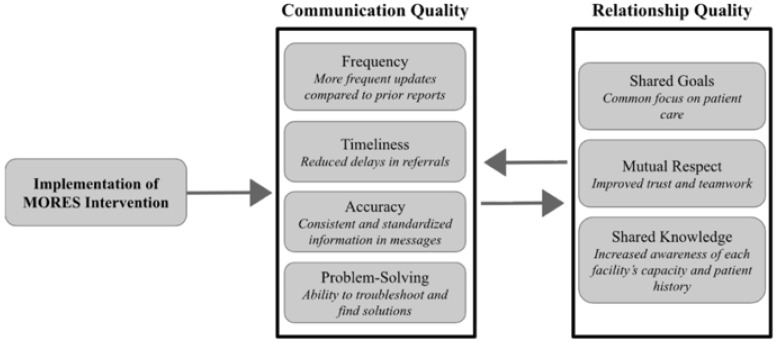
Organization of themes and sub-themes capturing the healthcare providers’ perspectives on how MORES influenced patient-related communication and provider–provider relationships using the relational coordination theory [19].

Using the relational communication theory, two themes and seven sub-themes were identified. Figure 1 presents the organization of the themes and sub-themes. The two main themes were identified as: (1) communication quality and (2) relationship quality. Communication quality maps on to the relational coordination theory construct of communication patterns which are based on respect, trust and shared goals and include the sub-themes frequency, timeliness, accuracy, and problem-solving. The qualitative data also supported a reporting of improved relationship quality (the second theme) amongst the RHF and hospital providers, including sub-themes of shared goals, mutual respect, and shared knowledge, which ultimately translated to increased trust and teamwork. The second theme aligns with the relational qualities construct of the theoretical framework which leads to timely, accurate problem-solving. Taken together, these themes support promptness and precision in fulfilling task-related clinical intervention reflecting effective collaboration and work coordination.

### 3.4. Communication Quality

Implementation of the MORES intervention enhanced the overall quality of communication between rural health facilities and district hospital providers. Participants reported that messages were shared more frequently and with greater timeliness compared to written referrals, reducing delays in the referral process. Communication also became more accurate through standardized information sharing, which minimized confusion and errors. Importantly, the MORES supported collaborative problem-solving by giving providers a platform to troubleshoot and identify solutions together, leading to smoother referral coordination.

#### 3.4.1. Frequency

Participants reported more frequent communication between facilities through the MORES intervention. Prior to the MORES, hospitals could not anticipate the patient’s arrival, as most communications were through paper referrals that were carried by the woman being referred or her family member. The MORES enabled RHF providers to update hospitals frequently as they were preparing and monitoring the referral process. “Whatever the delay [the RHF] were having, we continued to communicate through phone.” [P2012].

Frequent feedback was provided to the RHFs during the patient’s stay at the hospital as well: “As soon as you [the hospital provider] see the patient, you say [to the RHF provider] I received this patient, maybe the patient will go for surgery, or the patient has already delivered.” [P2053]. Then, after the patient receives treatment at the hospital, RHFs receive patient feedback from the hospitals, allowing for patient follow-up to occur:

“[With] the referral now, you know the patient’s condition already. So [the RHF will] check on them. Like a patient who came from Phebe and was supposed to come back after two weeks but did not come. Why are they not coming? Then we’ll tell the community health workers to inform them to come.”

[P1006]

This ensured that the RHF providers are constantly aware of the patient’s condition, establishing continuity of care, and allowing for the RHF providers to plan for follow-up care as needed.

#### 3.4.2. Timeliness

Reducing delays in referrals through timely communication enabled patients to receive more prompt care. By informing the hospital ahead of time regarding the patient’s diagnosis and estimated time of arrival, the hospital providers could prepare supplies and personnel to immediately treat the patient upon arrival, thereby reducing long wait times:

“When [the patient] comes… [with] a cord prolapse, you already know the management, you already have your gauze, NS (normal saline) set up… So the chance of that patient losing their baby [lowers], [since] prompt action has been taken based from the early information sent.”

[P2034]

Furthermore, the MORES established a new avenue for hospitals to easily refer patients to each other if there were inadequate staff or supplies before patient arrival. Prior to the MORES intervention, patients would arrive at a hospital and if there were not enough staff, no available operation room, or other resources were scarce, the patient would have to be referred again to a different hospital:

“Sometimes [CB Dunbar hospital] used to send patients to Phebe [hospital] without communication. And you will remain right in the ER and Phebe will send the patient back and telling you there is no doctor. So all that delay, it used to come from us as healthcare workers. But WhatsApp has helped us [because] when we send a text message that we have this patient at CB Dunbar but we don’t have these things. If Phebe has [those] materials, they will say, hey, don’t send the patient. We’re sending a car with the [needed] materials.”

[P2048]

The MORES intervention reduced the additional delays that happened while hospitals were figuring out which facility had resources and the staff to accept the referral. This additional communication loop also provided healthcare workers with more options, allowing them to refer patients to the best equipped facility in the first place.

#### 3.4.3. Accuracy

Having a consistent and standardized method of referral led to greater accuracy of the information sent back and forth during the referral process. Hospital providers knew the standard information expected to be included in the referral message, so if information was missing, they could clarify before patient arrival:

“Like if [the RHF provider] serve certain medication and there is no written time to know when to serve the next medication, it’s difficult to get to them [prior to MORES intervention]. But with WhatsApp, you can easily get back to them [and ask:] you served this medicine, what time did you give the medicine? You create that communication line between the clinic and the hospital.”

[P2032]

This ensured that providers had a more accurate understanding of the patient’s medical history and current condition before the patient arrives, leading to more effective care. Additionally, being able to ask for updated patient conditions ensured both RHF and hospital providers had the most accurate knowledge reflecting the patient’s most recent condition, thereby ensuring continuity of care.

#### 3.4.4. Problem-Solving

The MORES platform provided a new and easier avenue for healthcare providers to collaborate with each other to problem solve in an ongoing manner. Healthcare providers would often use the platform to share resources and information with each other to solve a medical problem one facility was facing. For instance, when one RHF expressed that they did not have the necessary supplies or manpower through WhatsApp, other RHFs would join to offer advice or support in looking for resources:

“Even in fact, when you’re calling for an ambulance, if there’s no ambulance available [at your facility], but then when [other RHF workers] have the idea to contact someone at another facility to get an ambulance, they will be able to help to call you. To give the ambulance contact number to you.”

[P1012]

Participants expressed that having the MORES intervention allowed timely communication across multiple facilities, leading to a faster resolution of the problem one facility was encountering. Hospitals would also communicate to share their expertise: “When [the RHF provider] referred a patient to CB Dunbar and doctors don’t have much knowledge about the case, they communicate with doctors at Phebe…” [P1014]. This ensured that the patient still received the care that they needed, and the hospitals were collaborating to solve problems together.

### 3.5. Relationship Quality

Beyond communication, the MORES was linked to improving professional relationships among healthcare providers. Shared goals around patient care became more explicit, fostering a collective sense of responsibility across facilities. Mutual respect was reinforced through greater trust and teamwork amongst providers, with providers recognizing each other’s contributions in the continuum of care. Additionally, the platform facilitated knowledge sharing by increasing awareness of each facility’s capacity and patient histories, which further promoted continuity of care and inter-facility collaboration.

#### 3.5.1. Shared Goals

Healthcare providers shared goals to provide quality care to their patients. Healthcare providers from both types of facilities discussed how the MORES connected them to more healthcare providers they had previously never talked to before, and how the shared goal of caring for the patient allowed them to connect deeply:

“Because we have our common interest, which is the patient, you will call me. I don’t have to know you personally, but you can call me and introduce yourself. Saying “this is the problem and we are sending the patient.” And I get prepared. And I get to know who I’m talking with.”

[P2037]

The deep bond between providers is reflected in the high median rating of 5.0 (IQR = 4.0–5.0) that RHF and hospital providers gave in response to the survey question “I feel like I am part of the healthcare team”. The shared goals of enhancing patient care also motivated healthcare providers to reach out to more healthcare professionals, thereby establishing a wider network of providers they could rely on to optimize the referral system: “If I call this person, and am unable to get the person, I can call a different person. Even if the person is at home, they will tell me, ‘let me call another person.’” That’s the connection.” [P1035]. Such benefit of the MORES was reflected in the median score of 5.0 (IQR = 4.0–5.0) to the survey question: “I feel like the use of WhatsApp improved referral communication”.

#### 3.5.2. Mutual Respect

Healthcare providers developed more respect for each other’s skills and workload, which led to greater trust and teamwork. When a referral was completed, the healthcare providers’ respect for each other’s clinical skills grew, further encouraging them to work together again:

“Yes, I feel connected because the person that will send this patient, I’ll already get that person’s contact and they will know me. So, if they send me another patient, they will call, ‘oh, you on shift?’ … They want to know if I’m on shift, because of the first patient, the one I dealt with, they will want to interact with me again. They say, ‘oh, I sent my patient to [this provider] and she was able to do one or two things for my patient, and my patient came through.’”

[P2052]

The increase in respect between providers was also demonstrated in the high median rating of 4.0 (IQR = 4.0–5.0) in response to the statement: “I feel respected by the providers from the hospital/rural health facility.” Healthcare providers gained a sense of trust in each other’s competency, reflected in the high median rating of 5.0 (IQR = 4.0–5.0) when asked if they respected the other group of providers. The MORES enabled participants to recognize each other’s competency.

“I adhere to [the rural midwife’s] advice and referred the patient… the doctor [at the hospital] said exactly the same thing the midwife said and thanked us for taking that brilliant step. I was impressed. Since then, my respect for her decisions has grown.”

[P1034]

The communication system required participants to rely on each other which heightened collaboration when treating patients.

#### 3.5.3. Shared Knowledge

Healthcare providers would often share knowledge with each other and learn from clinical cases together, which was possible due to the increased pathways of communication established through the MORES. When RHF providers referred a patient with an incorrect diagnosis, the hospital providers would reach out to explain and provide the correct diagnosis, which broadened RHF providers’ clinical skills:

“For example, I referred a patient because I suspected intrauterine fetal death using a pinard stethoscope. Not knowing that I wasn’t hearing the fetal heartbeat because it was lying on its back. So, I referred stating that the fetal was dead, but my suspicion was incorrect. The doctor who received the patient gave me feedback that my suspicion was incorrect and said next time I should consider using a Doppler.”

[P1000]

As communication improved, collaboration and knowledge sharing also increased, especially for the RHF providers. This increased trust and respect for each other’s clinical expertise was reflected in the score of regarding trust in each other’s clinical judgment and 4.0 (IQR = 4.0–5.0) in regard to confidence in each other (IQR = 4.0–5.0). Gaining more clinical skills from sharing knowledge with each other led to better patient care. Furthermore, health education would often be held in group chats with other healthcare providers, which encouraged discussion and learning:

“Yes, every one of us can be in the chat room talking about our conditions that we suspected. … Even get some conditions that we actually really never knew about. Like maybe when our friends see the same information that we saw. It can help you [in the future] when you see it in your own facility [and know that certain condition], it’s a danger sign.”

[P1003]

## 4. Discussion

The findings from our study suggest the MORES intervention was associated with a perceived improvement in communication and relationship qualities between RHF and hospital providers. Providers at both types of facility emphasized the MORES intervention allowed for frequent, timely, and accurate communication regarding patient conditions which further led to them having an increased sense of shared goals, mutual respect, and shared knowledge. Perceived improvement in communication quality led to the perception of stronger relationship qualities and vice versa.

Previous studies regarding the MORES intervention in Liberia have demonstrated that the bidirectional communication facilitated by the MORES led to faster referral times, increased coordination and accountability amongst providers, and increased patient satisfaction [15]. Interventions aiming to improve obstetric emergency referrals in sub-Saharan Africa facilitated greater communication amongst providers, led to increased provider satisfaction with the referral process, and more collaboration amongst providers [5]. A study on interprofessional collaboration amongst 35 healthcare workers at a secondary-level hospital in Ghana found that open communication and respect facilitated greater job satisfaction and more respectful delivery of maternal care [26]. As these studies demonstrate, the MORES is similar to other referral interventions in sub-Saharan Africa that were linked to an increase in communication and collaboration amongst providers. The association between bidirectional communication and improved patient care indicates that more health policies in LMICs should focus on increasing the reliability of clinical communication across healthcare facilities.

The MORES has been shown to be feasible and effective as an obstetric referral system with recommendation for scale-up across Liberia [27]. Similar interventions in other countries have yielded favorable results. A recent study in Ghana used the WhatsApp platform to link 13 health facilities and 81 healthcare providers to manage over 600 referrals from RHFs to district hospitals [28]. The study results displayed similar collaborative efforts to ours, with the WhatsApp messaging platform being used to conduct referrals and exchange clinical knowledge on treatment, leading to increased pre-referral treatment in facilities [28]. An intervention that established a phone-based obstetric referral system between an RHF and a district hospital in Uganda demonstrated similar results to the MORES, with increased feedback to the RHF to establish care continuity, more timely patient care upon arrival at the hospital, and increased motivation and connection amongst the healthcare workers [29]. Nevertheless, prior research has highlighted several persistent challenges to the implementation and scalability of the MORES, namely recurring data costs, inadequate network connectivity, and the demand for consistent, repeated training across all staff levels (cite: Reynold et al., JMIR paper). Despite these challenges, the MORES is overall perceived as feasible, acceptable, and scalable in other counties of Liberia and similar settings facing similar barriers.

### Strengths and Limitations

There are several limitations to this study. While the small sample size and geographical homogeneity limit the generalizability of the results outside of the current setting, our study also captured in-depth and fully contextualized understanding of the local health facilities. Additionally, our sample of healthcare professionals is unevenly distributed, with the majority of participants consisting of nurses and midwives. Recruiting a wider variety of healthcare professionals, such as doctors, paramedics and administrators, could reveal a different perspective to that held by the nurses and midwives. However, it is the nurses and the midwives making the referrals in these settings, justifying the limited diversity of healthcare professionals. Furthermore, selection bias may have affected the results, as the sample of healthcare providers was recruited through word-of-mouth. Most of the participants were also involved in the pilot intervention and may be more inclined to evaluate the project more favorably, leading to social desirability bias. The ceiling effect could have occurred based on the use of the Likert scale, but we provide the median to report sensitivity.

## 5. Conclusions

When assessed one year after completing the MORES pilot intervention, healthcare providers perceived an increase in shared goals, mutual respect, and connection with each other through the communication and collaboration. As seen through the relational communication framework [20], healthcare providers perceived the MORES to have a positive effect on communication quality as it facilitated frequent and timely patient updates, improved accuracy of shared information, and increased collaboration to solve problems. This led to the development of shared motivation, an increased sense of trust and teamwork, and more knowledge sharing to broaden clinical skill sets, especially amongst RHF providers. Of note, the findings from this study demonstrate that the MORES remains effective in facilitating collaboration between different healthcare facilities, while prior studies have demonstrated its success in reducing cesarean section wait times and improved neonatal outcomes [2]. These findings inform policymakers, clinicians, and researchers intending to implement a technology-based obstetric referral system in similar low-resource settings. Based on our study results, the MORES intervention has been recommended for scale-up by local stakeholders [27]. Further research is needed to identify the context-specific barriers and facilitators in other settings across Liberia and beyond.

## Figures and Tables

**Table 1:** Demographic characteristics and participation in previous MORES study across participating healthcare providers.

	Total	Rural Health Facility	Hospital	*p*-Value
Total	N = 91	N = 35	N = 56	
Age, mean (SD) ^a^	41.3 (7.4)	38.3 (5.9)	43.2 (7.7)	0.002 **
Female	82 (90.1%)	32 (91.4%)	50 (89.3%)	
Male	9 (9.9%)	3 (8.6%)	6 (10.7%)	
Nurse	27 (29.7%)	9 (25.7%)	18 (32.1%)	0.51
Midwife	60 (65.9%)	26 (74.3%)	34 (60.7%)	0.006 **
Physician assistant	4 (4.4%)	0 (0.0%)	4 (7.1%)	0.11
How many years have you been in your profession? ^a^	9.5 (3.8)	8.3 (3.6)	10.4 (3.8)	0.010 **
How many years have you been at your current location? ^a^	6.9 (3.8)	5.2 (3.6)	8.0 (3.7)	<0.001 ***
Do you have a personal phone that can send text messages?				0.15
Yes	88 (96.7%)	33 (94.3%)	55 (98.2%)	
No	2 (2.2%)	2 (5.7%)	0 (0.0%)	
Prefer not to answer	1 (1.1%)	0 (0.0%)	1 (1.8%)	
Is it a smartphone?				0.26
Yes	87 (95.6%)	32 (91.4%)	55 (98.2%)	
No	3 (3.3%)	2 (5.7%)	1 (1.8%)	
Prefer not to answer	1 (1.1%)	1 (2.9%)	0 (0.0%)	
Do you use WhatsApp?				0.16
Yes	79 (86.8%)	32 (91.4%)	47 (83.9%)	
No	11 (12.1%)	2 (5.7%)	9 (16.1%)	
Prefer not to answer	1 (1.1%)	1 (2.9%)	0 (0.0%)	
Do you use your phone every day?				0.19
Yes	89 (97.8%)	33 (94.3%)	56 (100.0%)	
No	1 (1.1%)	1 (2.9%)	0	
Prefer not to answer	1 (1.1%)	1 (2.9%)	0	
Were you part of the original WhatsApp study?				0.17
Yes	84 (92.3%)	34 (97.1%)	50 (89.3%)	
No	7 (7.7%)	1 (2.9%)	6 (10.7%)	
If yes, are you working in the same location as you were at the start of the study?				0.24
Yes	79 (86.8%)	30 (85.7%)	49 (87.5%)	
No	4 (4.4%)	3 (8.6%)	1 (1.8%)	
Prefer not to answer	8 (8.8%)	2 (5.7%)	6 (10.7%)	
Did you use the WhatsApp program for the referral of pregnant women?				0.27
Yes	75 (82.4%)	30 (85.7%)	45 (80.4%)	
No	4 (4.4%)	0 (0.0%)	4 (7.1%)	
Prefer not to answer	12 (13.2%)	5 (14.3%)	7 (12.5%)	

^a^ Conducted independent *t*-test, remainder were chi-square tests. ** *p*-value ≤ 0.01, *** *p*-value ≤ 0.001.

**Table 2:** Trust and Teamwork Survey results from participating healthcare providers.

	Total Mean (SD); Median (IQR)	Rural Health Facility Mean (SD); Median (IQR)	Hospital Mean (SD); Median (IQR)	*p*-Value
I respected providers from the hospital/rural health facility	4.6 (0.5); 5.0 (4.0–5.0)	4.4 (0.5); 4.0 (4.0–5.0)	4.6 (0.5); 5.0 (4.0–5.0)	0.046 *
I feel respected by the providers from the hospital/rural health facility	4.4 (0.7); 4.0 (4.0–5.0)	4.1 (0.9); 4.0 (4.0–5.0)	4.6 (0.5); 5.0 (4.0–5.0)	0.004 **
I feel a bond with providers from the hospital/rural health facility	4.2 (0.8); 4.0 (4.0–5.0)	4.1 (0.9); 4.0 (4.0–5.0)	4.3 (0.7); 4.0 (4.0–5.0)	0.27
I have confidence in providers from the hospital/rural health facility	4.3 (0.6); 4.0 (4.0–5.0)	4.5 (0.6); 4.0 (4.0–5.0)	4.1 (0.6); 4.0 (4.0–5.0)	0.007 **
I trust the clinical judgment of the providers in the hospital/rural health facility	4.1 (0.6); 4.0 (4.0–5.0)	4.4 (0.6); 4.0 (4.0–5.0)	3.9 (0.6); 4.0 (4.0–5.0)	<0.001 ***
I feel like I am part of a healthcare team	4.9 (0.3); 5.0 (4.0–5.0)	4.7 (0.5); 5.0 (4.0–5.0)	5.0 (0.1); 5.0 (4.0–5.0)	<0.001 ***
I feel like my experience is recognized by my peers	4.8 (0.4); 5.0 (4.0–5.0)	4.7 (0.5); 5.0 (4.0–5.0)	4.8 (0.4); 5.0 (4.0–5.0)	0.23
I feel like the use of WhatsApp improved referral communication	4.9 (0.3); 5.0 (4.0–5.0)	4.9 (0.3); 5.0 (4.0–5.0)	4.9 (0.3); 5.0 (4.0–5.0)	0.88
I feel like providers at the hospital/rural health facility are an obstacle to my work	1.8 (0.4); 2.0 (2.0–2.0)	1.7 (0.4); 2.0 (2.0–2.0)	1.8 (0.4); 2.0 (2.0–2.0)	0.64
Receiving patient feedback improves my clinical skills/Receiving advanced knowledge of a referral helps me prepare for the patient’s arrival	4.9 (0.4); 5.0 (4.0–5.0)	4.7 (0.6); 5.0 (4.0–5.0)	5.0 (0.1); 5.0 (4.0–5.0)	<0.001 ***

Note: SD: standard deviation; IQR: interquartile range; the *p*-values were calculated using the Mann–Whitney U test. There were two versions of these questions, one for the providers at the rural healthcare facility asking about providers at the hospital and vice versa. The response options are rated on a Likert scale of 1–5, 1 meaning no confidence/disagreement with the statement and 5 meaning full confidence/strongly agree with the statement. * *p*-value ≤ 0.05, ** *p*-value ≤ 0.01, *** *p*-value ≤ 0.001.

## Data Availability

The original contributions presented in this study are included in the article. Further inquiries can be directed to the corresponding authors. Public deposition of the data would breach compliance with the protocol approved by the research ethics board, specifically the participant consent. Upon reasonable request, the data may be available.

## Abbreviations

The following abbreviations are used in this manuscript:

RHF	Rural health facility
BEmONC	Basic emergency obstetric and newborn care services
CEmONC	Comprehensive emergency obstetric and newborn care services
MORES	Mobile obstetric referral system
mHealth	Mobile health
II	Individual interviews
RA	Research assistants

## References

[1] WHO (2025). Maternal Mortality. https://www.who.int/news-room/fact-sheets/detail/maternal-mortality.

[2] Lee H., Dahn B., Sieka J., Nyanplu A., Reynolds C.W., Edson C., Lockhart N., Lori J.R. (2024). The use of a mobile obstetric emergency system to improve obstetric referrals in Bong County, Liberia: A pre-post study. Int. J. Gynaecol. Obstet..

[3] Chavane L.A., Bailey P., Loquiha O., Dgedge M., Aerts M., Temmerman M. (2018). Maternal death and delays in accessing emergency obstetric care in Mozambique. BMC Pregnancy Childbirth.

[4] Lee H., Perosky J., Horton M., Reynolds C., Nyanplu A., Lori J.R. (2023). Verbal autopsy analysis of maternal mortality in Bong County, Liberia: A retrospective mixed methods study. BMJ Open Qual..

[5] Avoka C.K., McArthur E., Banke-Thomas A. (2022). Interventions to improve obstetric emergency referral decision making, communication and feedback between health facilities in sub-Saharan Africa: A systematic review. Trop. Med. Int. Health.

[6] Geleto A., Chojenta C., Musa A., Loxton D. (2018). Barriers to access and utilization of emergency obstetric care at health facilities in sub-Saharan Africa: A systematic review of literature. Syst. Rev..

[7] Kim J., Barreix M., Babcock C., Bills C.B. (2017). Acute Care Referral Systems in Liberia: Transfer and Referral Capabilities in a Low-Income Country. Prehosp. Disaster Med..

[8] King J., Tarway-Twalla A.K., Dennis M., Twalla M.P., Konwloh P.K., Wesseh C.S., Tehoungue B.Z., Saydee G.S., Campbell O., Ronsmans C. (2022). Readiness of health facilities to provide safe childbirth in Liberia: A cross-sectional analysis of population surveys, facility censuses and facility birth records. BMC Pregnancy Childbirth.

[9] El-Sherif D.M., Abouzid M. (2022). Analysis of mHealth research: Mapping the relationship between mobile apps technology and healthcare during COVID-19 outbreak. Glob. Health.

[10] McCool J., Dobson R., Whittaker R., Paton C. (2022). Mobile Health (mHealth) in Low- and Middle-Income Countries. Annu. Rev. Public Health.

[11] Atnafu A., Otto K., Herbst C.H. (2017). The role of mHealth intervention on maternal and child health service delivery: Findings from a randomized controlled field trial in rural Ethiopia. mHealth.

[12] Dzomeku V.M., Mensah A.B.B., Nakua E.K., Agbadi P., Okyere J., Kumah A., Munukpa J., Ofosu A.A., Lockhart N., Lori J.R. (2023). Feasibility of the use of WhatsApp messaging technology to facilitate obstetric referrals in rural Ghana. BMC Digit. Health.

[13] McNabb M., Chukwu E., Ojo O., Shekhar N., Gill C.J., Salami H., Jega F. (2015). Assessment of the quality of antenatal care services provided by health workers using a mobile phone decision support application in northern nigeria: A pre/post-intervention study. PLoS ONE.

[14] Nardo B., Cannistrà M., Diaco V., Naso A., Novello M., Zullo A., Ruggiero M., Grande R., Sacco R. (2016). Optimizing patient surgical management using WhatsApp application in the Italian healthcare system. Telemed. e-Health.

[15] Reynolds C.W., Lee H., Sieka J., Perosky J., Lori J.R. (2024). Implementation of a Technology-Based Mobile Obstetric Referral Emergency System (MORES): Qualitative Assessment of Health Workers in Rural Liberia. JMIR mHealth uHealth.

[16] Reynolds C., Horton M., Lee H., Harmon W., Sieka J., Lockhart N., Lori J.R. (2023). Acceptability of a WhatsApp triage, referral, and transfer system for obstetric patients in rural Liberia. Ann. Glob. Health.

[17] Bispo Júnior J.P. (2022). Social desirability bias in qualitative health research. Rev. Saude Publica.

[18] Lee H., Kim S., Sieka J., Harmon-Gray W.-M., Veliz P.T., Lori J.R. (2025). Improving decision-to-incision interval (Ddi) of emergency cesarean sections through mobile-based obstetric emergency system (Mores) and midwife-led triage in bong county, liberia: A quasi-experimental study. Int. J. Environ. Res. Public Health.

[19] Gittell J.H. (2002). Coordinating Mechanisms in Care Provider Groups: Relational Coordination as a Mediator and Input Uncertainty as a Moderator of Performance Effects. Manag. Sci..

[20] Liberia World Bank Gender Data Portal. n.d. https://genderdata.worldbank.org/en/economies/liberia.

[21] Liberia Ministry of Internal Affairs, Ministry of Finance and Development Planning (2025). Bong County Development Agenda.

[22] Lori J.R., Munro M.L., Moore J.E., Fladger J. (2013). Lessons learned in Liberia: Preliminary examination of the psychometric properties of trust and teamwork among maternal healthcare workers. BMC Health Serv. Res..

[23] Editorial Team (2006). Liberia: Language situation. Encyclopedia of Language & Linguistics.

[24] Jowsey T., Deng C., Weller J. (2021). General-purpose thematic analysis: A useful qualitative method for anaesthesia research. BJA Educ..

[25] Glaser B.G. (1965). The constant comparative method of qualitative analysis. Soc. Probl..

[26] Dzomeku V.M., Dassah E., Gyimah E.M., Emikpe A.O., Owusu L.B., Dwumfour C.K., Ogunyewo O.A., Agyekum T.P., Boadu E.A., Nakua E.K. (2025). Providers perspectives on a team-based maternal health care delivery in Ghana: A qualitative study. PLoS Glob. Public Health.

[27] US Agency for International Development (2024). Scaling Up a Mobile Obstetric Triage and Referral System in Liberia Improves Maternal Outcomes.

[28] Owen M.D., Ismail H.M., Goodman D., Batakji M., Kim S.M., Olufolabi A., Srofenyoh E.K. (2022). Use of WhatsApp messaging technology to strengthen obstetric referrals in the Greater Accra Region, Ghana: Findings from a feasibility study. PLoS ONE.

[29] Kanyesigye H., Ngonzi J., Mulogo E., Fajardo Y., MacDonald N.E., Kabakyenga J. (2023). Acceptability of phone-based communication intervention by healthcare workers as an adjunct to routine referral form: A qualitative study in south western uganda. Open J. Prev. Med..

